# Railway Crossing Risk Area Detection Using Linear Regression and Terrain Drop Compensation Techniques

**DOI:** 10.3390/s140610578

**Published:** 2014-06-16

**Authors:** Wen-Yuan Chen, Mei Wang, Zhou-Xing Fu

**Affiliations:** 1 Department of Electronic Engineering, National Chin-Yi University of Technology 57, Sec. 2, Zhongshan Rd., Taiping Dist., Taichung 41170, Taiwan; E-Mail: wangm@xust.edu.cn; 2 College of Electric and Control Engineering, Xi'an University of Science and Technology, 58 Yan-Ta Road, Xi'an City 710054, Shaanxi Province, China; E-Mail: fuzx@xust.edu.cn

**Keywords:** railway crossing, object extraction, background subtraction, linear regression

## Abstract

Most railway accidents happen at railway crossings. Therefore, how to detect humans or objects present in the risk area of a railway crossing and thus prevent accidents are important tasks. In this paper, three strategies are used to detect the risk area of a railway crossing: (1) we use a terrain drop compensation (TDC) technique to solve the problem of the concavity of railway crossings; (2) we use a linear regression technique to predict the position and length of an object from image processing; (3) we have developed a novel strategy called calculating local maximum Y-coordinate object points (CLMYOP) to obtain the ground points of the object. In addition, image preprocessing is also applied to filter out the noise and successfully improve the object detection. From the experimental results, it is demonstrated that our scheme is an effective and corrective method for the detection of railway crossing risk areas.

## 1. Introduction

Nowadays there are six kinds of railway crossings as classified by Taiwan Railroad Affairs: (1) the first kind of railway crossing has an isolator and alarm device, and it is watched by a workman; (2) the second kind of railway crossing has an isolator and alarm device, and it is watched by a workman on a regular schedule; (3) the third kind of the railway crossing has an isolator and alarm device, but it is not watched by anyone; (4) the fourth kind of the railway crossing is also called as a half-close type, and it is similar to type three. It only provides 1.5 m of space for pedestrians or motorcycles to pass; (5) the fifth kind of the railway crossing has an isolator and alarm device, and it is controlled by a human only when a train is passing; (6) the sixth kind of the railway crossing is a special purpose railway crossing. This kind of railway crossing is built for a particular company and therefore, it is controlled by that company.

In America, railway crossings are classified according to their traffic control facilities. There are two distinct types: one is an active crossing where all the facilities are controlled by the train's approach. Once the train is approaching, the system will enable all the alarm and facilities automatically. The other is a passive crossing: all the alarm systems and facilities are controlled by a human when a train is approaching. It offers the operator control of all the facilities when the train is approaching.

In pace with technology progress, several transport facilities have been improved. Lots of sensors used to detect the environment are now applied to transport facilities for people's safety. In the world, many active sensors are being developed for improving railway crossing safety; including induced coil, infrared, wide-band radar, and ultrasonic sensors. However, there still exist some problems, for example, dead angles in the detection, climate, the wind direction, interference with the environment.

Several authors have proposed lots of safety measures for railway crossings based on different sensor techniques. Sato [1] developed a method using ultrasound to detect an obstacle. Lohmeier [2] presented an obstacle-detecting method using a radar technique. Takeda [3] proposed a method for improving the signals of a railway crossing. Ku [4] designed a set of devices to show in meters how close the train is. Besides, many authors [5–11] have proposed methods for improving the safety of railway crossings using image detection techniques.

Han and Lee [12] presented the palm vein texture concept and applied a texture-based feature extraction method to achieve the palm vein authentication. In their algorithm, Gabor filters were used to achieve the optimized resolution in both the spatial and frequency domains. For obtaining an effective palm vascular pattern, they adopted a bit string by coding the palm vein features using an innovative and robust adaptive Gabor filter method. Meanwhile, two VeinCodes were measured by a normalized Hamming distance technique. Finally, they got highly accurate and rapid real-time palm vein recognition. From the simulation results, it is demonstrated that their method is feasible and effective in the palm vein recognition. Other methods [13–14] have been presented for the object detection by using image processing techniques.

In this research, we define that the risk area as the interval between the railway crossing gates. Further, we detect the railway crossing risk area by image processing techniques. Once humans intrude, the system will automatically emit an alarm and send a signal to the driver to stop the train. The remainder of this paper is organized as follows: Section 2 shows the related work. Section 3 shows the system algorithm. Section 4 describes the experimental results. Finally, Section 5 presents the conclusions of this paper.

## 2. Related Work

Measuring an object's distance and size in an image usually uses a linear regression technique because it is an easier and more effective computing method wherever the object is located. However, the ground of the object is located on is not always flat, so terrain drop compensation is necessary.

### 2.1. Linear Regression

**Figure 1.**
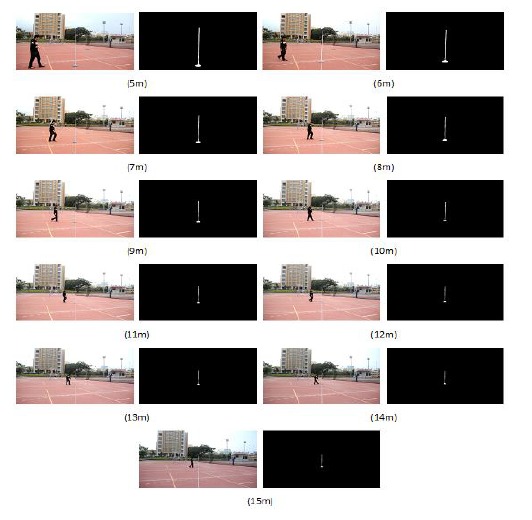
The test images; the objects height shows a linear relation corresponding to the distance between the rod and camera when the rod is varied from 5 m to 15 m.

**Figure 2.**
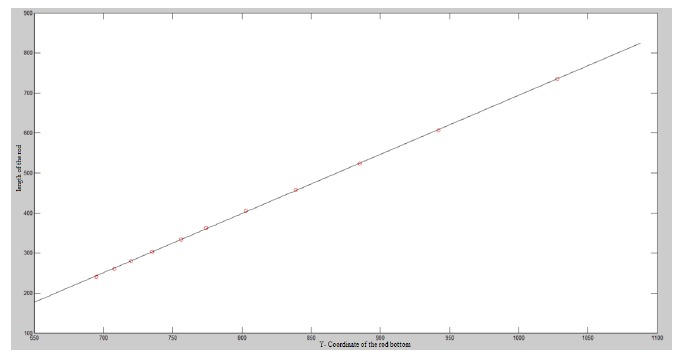
The rod length in the image corresponding to the distance between the rod and camera.

Figure 1 shows the images which are used to construct a linear relation corresponding to the distance between the rod and camera. Since a curve makes it easier to show the linear relationship, we draw a curve using the test data as shown in Figure 2, where the X-coordinate denotes the length of the rod, and the Y-coordinate expresses the location of the bottom of the rod. From the figure we can clearly see that it is a linear relationship.

In this paper, we use the least square method to construct the linear regression and get the formula *l* = *ap* + *b*. The parameters are listed below:

(1) l=ap+b

(2) $$\bar{p}=\frac{\overset{n}{\underset{i=1}{\text{∑}}}p_{i}}{n}$$

(3) $$\bar{l}=\frac{\overset{n}{\underset{i=1}{\text{∑}}}l_{i}}{n}$$

(4) $$a=\frac{\overset{n}{\underset{i=1}{\text{∑}}}\left(p_{i}-\bar{p})(l_{i}-\bar{l}\right)}{\overset{n}{\underset{i=1}{\text{∑}}}{\left(p_{i}-\bar{p}\right)}^{2}}$$

(5) $$b=\bar{l}-a\bar{p}$$

where *l* is the altitude of the railway crossing gates, meaning the height of the gates to the ground, *p* denotes the location of the gates' projection to the ground, *l̄* expresses the average of all the *l_i_*, and *p̄* is the mean of all the *p_i_*, *a* is the first order coefficient of the linear equation *l* = *ap* + *b*, and *b* is the constant term of the equation *l* = *ap* + *b*.

### 2.2. Terrain Drop Calculation

Terrain drop is caused by the difference between the height of some point and the standard horizontal line. In the detection of a railway crossing's risk area, we need to calculate the terrain drop used as an offset for different uneven pavements. In this study case, the terrain drop is the object length difference in the image between the camera mounted on a horizontal line and the road. For calculating the terrain drop, several conditions of the camera need to be set. First we need to find out the drop level. It means that we measure an object height at a railway crossing and the flat ground, and then recode its difference as the offset of the terrain drop compensation. In this stage, we set the camera up under the same conditions such as the angle up or down, the focus of the camera, the same object, the same object length and at the same picture taking distance.

**Figure 3.**
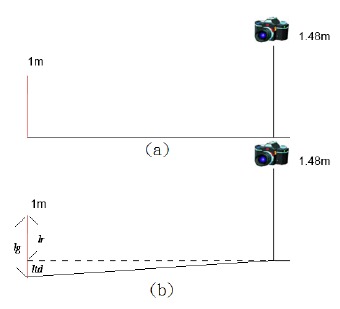
The object photography schematic diagram: (**a**) image captured by the camera on flat ground; (**b**) image captured by the camera on the railway crossing with a terrain drop problem.

Figure 3 shows the object photography schematic diagram. Figure 3a displays the relationship between the object and camera which is mounted on flat ground. On the contrary, Figure 3b expresses the case where the object length in an image is captured by a camera mounted on the railway crossing, and it has a terrain drop problem. This case reveals the object is shrinking the length at the protruding pavement of the railway crossing. The terrain drop calculation formula is shown in Equation (6):

(6) $$l_{td}=l_{g}-l_{r}$$

Where the *l_td_* is the value of the terrain drop compensation. *l_g_* expresses the altitude of the testing rod on flat ground measured by the camera, which is obtained by replacing the measurement data in Equation (1). On the other hand, *l_r_* denotes the altitude of the testing rod on the railway crossing measured by the camera.

## 3. Risk Area Detection Algorithm

**Figure 4.**
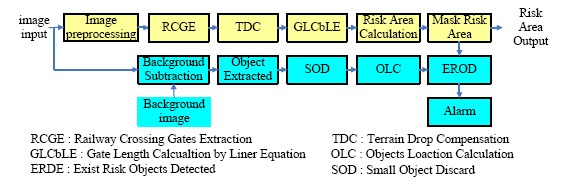
The flow chart of the railway crossing risk area detection.

Most railway accidents happen in the railway crossing neighborhood; therefore it is called the risk area. This section describes the risk area detection algorithm. Figure 4 shows the flow chart of the railway crossing risk area detection. In the detection procedure, first, an image preprocessing stage is used to filter out the noise and some processing is performed for improving the gate extraction. Next, the railway crossing gates extraction stage is used to get the gates' position. Since the area of the vertical projection of the gates is the risk area, the gates must be extracted. Furthermore, since the railway crossing always has the terrain drop problem, terrain drop compensation (TDC) is needed to correct the measurements. After TDC, the system can obtain the real altitude of the gates from the gate length calculation by a linear equation. Finally, the gates and their altitude are obtained, and then the risk area is calculated. Besides, the background subtraction method is used to extract the objects. Since the small object size can be treated as noise, we discard the small objects to filter them out. Furthermore, the objects location calculation (OLC) is used to compute the real position of the objects standing on the ground in the picture. After the OLC operation, we compare the ground position of the objects with the risk area of railway by the existing risk objects detection (EROD). If the result of the EROD is true, an alarm will be issued. The details of the operation can be found in Figure 4.

### 3.1. Image Preprocessing

**Figure 5.**
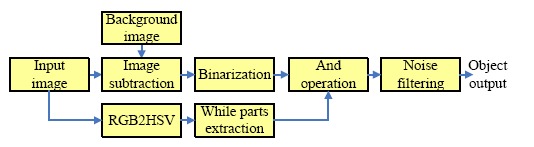
The flow chart of the image preprocessing of the risk area extraction.

The image preprocessing stage is used to filter out the noise to improve the object detection. Figure 5 shows the flow chart of the image preprocessing of the risk area extraction. Since a binary image is suitable for object extraction, a binarization stage was used to convert the object image into a binary form.

**Figure 6.**
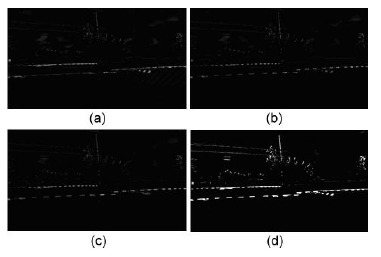
Background subtraction results in RGB planes: (**a**) the result of the background subtraction in the R plane; (**b**) the result of the background subtraction in the G plane; (**c**) the result of the background subtraction in the B plane; (**d**) the result after the summation and binarization of Figure 6b,c.

The input image and background image were used to extract the object image using an image subtraction operation. In fact, railway crossing gates in Taiwan are red alternating with white. After trial-and-error methods, we discovered the white part is suitable for used in extracting the gates. Thus, a white part extraction stage is an important part of the process. Simultaneously, a binarization operation was used to convert the grayscale images R, G, and B into binary images, respectively. Then, the AND operation was used to combine the three binary images and obtain a hybrid binary image. Accordingly the hybrid image value is high; it can map and store the input image to obtain the candidate parts of the gates. After several operation stages, such as white color part extraction, area masking, rotate right operation, closing operation, and length filtering, we obtained the final output. Figure 6 shows the background subtraction results in the different RGB planes. Figure 6a is the result of the background subtraction in the R plane, Figure 6b denotes the result of the background subtraction in the G plane, and Figure 6c expresses the result of the background subtraction in the B plane. Finally, Figure 6d is the result after the summation and binarization of Figure 6b,c.

Since the red upper-part of the gates will be changed to white in the sunshine, we select the white part of the gates to avoid the interference. Besides, the HSV color planes are suitable for the extracted white part. A RGB to HSV transfer is used to complete the goal. In the paper, we set the thresholds S < 0.3 and V > 0.5 used as the conditions for extracting the white parts. The relative formula is given in Equation (7):

(7) $$\begin{array}{c} S<0.3\\ V>0.5 \end{array}$$

### 3.2. The Gates Extraction

**Figure 7.**
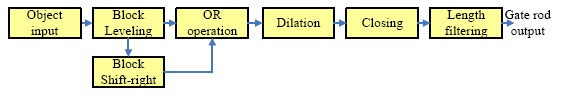
The flow chart of the railway crossing gates extraction.

**Figure 8.**
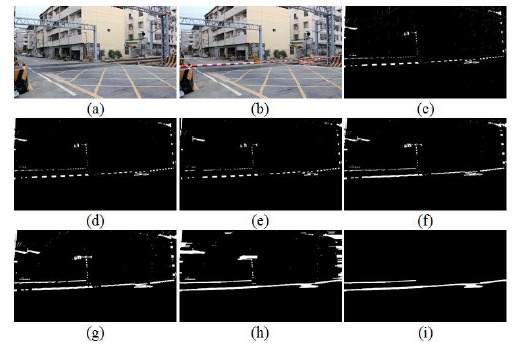
The gates extraction process: (**a**) the background image; (**b**) input test image; (**c**) white part extraction; (**d**) after filtering the noise; (**e**) white part rotation right of (d); (**f**) after merging (d) and (e); (**g**) after dilation operation; (**h**) after the closing operation; (**i**) after lengthy fitting operations.

Figure 7 shows the flow chart of the railway crossing gates extraction. In gates extraction, we develop a method called a white part associated with topology technique (WPAT) to detect the gate image parts. The gates image appears as several bar blocks like a dish bar after image preprocessing. In order to eliminate the dish bar appearance, first, we rotate right some of the bar block according to its length. Then the two images are summed; finally, the images before and after rotation are used to obtain a gate image without a dish. The result of this action generates an effect cascading the bar into a line. Figure 8 shows the railway gates extraction process. It shows the results of the block rotation right operation and cascading the gate rod as a line. Figure 8a is the background image, and Figure 8b is an input test image. Figure 8c shows the results of the white part extraction, and Figure 8d shows the results after filtering the noise. Figure 8e displays the results after white part rotation right of Figure 8d. Figure 8f displays the results after merging the images of Figure 8d,e. Successively, Figure 8g shows the results after dilation operation of Figure 8f,h shows the result after closing operation of Figure 8g. Finally, Figure 8i shows the result after lengthy fitting operations, it obviously cascades the white blocks as a line.

### 3.3. Terrain Drop Compensation

Since the railway maybe higher or lower than the nearby area, the railway crossing gates have the terrain drop problem. In order to solve the problem, terrain drop compensation (TDC) is used to improve the precision of the risk area calculation. In practice, the values of the TDC need to be obtained before the railway crossing risk area detection. Meanwhile, the data must come from the same camera image-grab conditions such as the angle up or down, the focus of the camera, the same test object and its length, and at the same distance to take a picture. From Equation (6) *l_td_* = *l_g_* − *l_r_*, we know the terrain drop compensation is *l_td_*, which needs to be obtained before starting the risk area detection, and *l_g_* and *l_r_* are the measured altitudes of the testing rod on flat ground and on the railway crossing under the same camera image-grab conditions. Besides, the length unit ‘cm’ and pixels conversion are also obtained.

**Figure 9.**
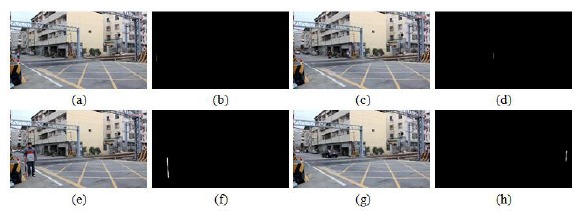
The test object with length 100 cm for the terrain drop compensation: (**a**) the test rod on rear-left; (**b**) the extracted test rod image of (a); (**c**) the test rod on rear-right; (**d**) the extracted test rod image of (c); (**e**) the test rod on front-left; (**f**) the extracted test rod image corresponding to (e); (**g**) the test rod on front-right; (**h**) the extracted test rod image corresponding to (g).

In order to obtain the terrain drop value, a real datum by image test is executed. We set the test rod (100 cm) on the four corners: rear-left, rear-right, front-left, and front-right of the railway crossing gate, the TDC is calculated before the risk area detection. Figure 9 shows the measurement process of the terrain drop. Figure 9a is the test rod on rear-left, and Figure 9b is the extracted test rod image of Figure 9a. Similarly, Figure 9c, e and g are the test rod images on the rear-right, front-left and front-right, respectively. Figure 9d, f and h are the extracted length images of those test rods by the image processing, respectively. Actually, the risk area is the quadrilateral area of the vertical projection of the railway crossing gates. In the other words, the risk area is constructed by the test rod length of the four corner images, which are the ground points of the gate projection.

### 3.4. Risk Area Detection

The risk area of the railway crossing is a quadrilateral, so we need to compute the four corners of the railway crossing gates first. In the other words, we need four sets of offset data used to calculate the quadrilateral. Here we use a rod with 100 cm length to generate the offset data. Figure 9 shows the compensation data calculation method of the four corners. According to Figure 9, all the data obtained is shown in Table 1. The parameters in Table 1 are defined in the following: in the measured test rod altitude *l_r_* (cm) item, the parameters ‘a’, ‘b’, ‘c’ and ‘d’ are expressed as the measured altitudes of the rod on the left-rear, left-front, right-front and right-rear corners, respectively. In terrain the drop *l_td_* item, the parameters ‘a’ , ‘b’, ‘c’, ‘d’ are expressed as the measured terrain drop of the rod positions on left-rear, left-front, right-front and right-rear, respectively. In the risk area *RA_S_* item, the parameters ‘a’ , ‘b’, ‘c’, ‘d’ are expressed as the measured segment length of the risk area from left-front point to left-rear point, from right-front point to right-rear point, from left-front point to right-front point and from left-rear point to right-rear point, respectively. Finally, in the errors *err_S_* item, the parameters ‘a’ , ‘b’, ‘c’, ‘d’ are expressed as the measured length difference of the risk area between the real measured and proposed calculated from left-front point to left-rear point, from right-front point to right-rear point, from left-front point to right-front point and from left-rear point to right-rear point, respectively.

**Figure 10.**
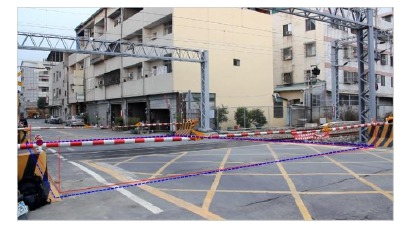
The detection result of the risk area, where the blue-line area is the detection result and the red-line area is the real measured result.

In Table 1, where the extracted test rod length on left-rear is 84 cm, on left-front is 88 cm, on right-front is 92 cm, and on right-rear is 85 cm, respectively, where the corresponding terrain drops are 21 cm, 25 cm, −1 cm, and −20 cm. After a risk area is calculated, the coordinates of the risk area are 80 cm, 64 cm, 278 cm and 117 cm. When the real measurement is compared and predicted by our method, the error cm of the four corners are 1 cm, 9 cm, 26 cm and 3 cm. From the results; it is evident that our scheme is effective and precise. Figure 10 shows the test results, where the blue-line area denotes the calculated risk area in our method, and the red-line area express the real measured risk area. It is obvious that the two colored lines are very close, and it means the error is small.

### 3.5. Local Y-coordinate Maximum Points

**Figure 11.**
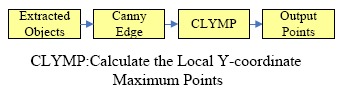
The flowchart of calculating the local Y-coordinate maximum points.

We have developed a novel strategy called calculating the local maximum Y-coordinate object points (CLMYOP). Since the original point of the picture is located in the left-up position, the ground points of the object must be on the local maximum Y-coordinate points. According to the CLMYOP approach, we can easily obtain the ground points of the objects. Figure 11 shows the flowchart of the search for object ground points. For an object, its ground points must be the points where the Y-coordinate is a local maximum, therefore, canny edge detection can be used to get the edge of the object. We successfully extract all the local maximum Y-coordinate object points on the edge. Certainly, if the one of CLMYOP points is located on the range of the railway risk area, the system must issue an alarm.

## 4. Experimental Results

### 4.1. Environmental Conditions

Several (1920 × 1088) test images are used in our simulation to demonstrate the performance of the proposed scheme. As the hardware environment, we use a personal computer equipped with a INTEL(R) Core(TM) i5 CPU under a 3.1 GHz system clock, 8 Gigabyte memory, and an AMD Radeon™ display card. Besides, we use the Matlab R2012a as the program developing system.

In order to let the linear regression technique be used in this project, we did several tests as shown in Section 2.1. The results show that the distance ranges of the camera to the railway from 5 to 15 m are good, and the camera angles from 15° to −15° are suitable. However, the data was obtained with the camera placed at a height of 1.48 m.

On the other hand, the risk area *RA_f_*_50_ and its errors *err_f_*_50_ with the camera moving forward 50 cm, and the risk area *RA_b_*_50_ and its error *err_b_*_50_ when the camera is moved back 50 cm are important tests that can demonstrate the effectiveness of the scheme.

### 4.2. Experimental Results

**Figure 12.**
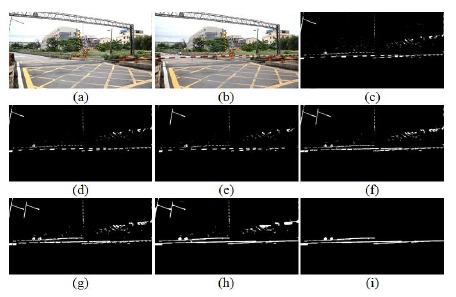
The gates extraction case-1, the railway crossing of the Doudile Road: (**a**) the background image; (**b**) the input test image; (**c**) the image after extracting white part; (**d**) the image after filtering the noise; (**e**) white part rotation right of (d); (**f**) the image after merging (d) and (e); (**g**) the image after dilation operation, (**h**) the image after the closing operation, (**i**) the image after lengthy fitting operations.

**Figure 13.**
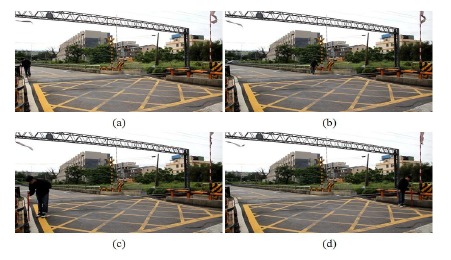
The terrain drop compensation measure of the railway crossing of the Doudile Road: (**a**) the rear-left corner; (**b**) the rear-right corner; (**c**) the front-left corner; (**d**) the front-right corner.

In order to demonstrate our method is effective, several railway crossing images and real-stat measurements are used for simulations. Figure 12 is the gates extraction case-1, the railway crossing image is taken from the Doudile Road. Figure 12a shows the background image, and Figure 12b shows the input test image. Figure 12c shows the resulting image after extracting the white part and Figure 12d expresses the resulting image after filtering the noise. Figure 12e denotes the resulting image after rotating right the white part of Figure 12d, f denote the resulting image after merging Figure 12d,e. Finally, Figure 12g shows the resulting image after the dilation operation, Figure 12h shows the resulting image after the closing operation, and Figure 12i displays the resulting image after lengthy fitting operations. On the other hand, Figure 13 shows the terrain drop compensation measure steps of the railway crossing of Doudile Road. Figure 13a–d show the rear-left corner, the rear-right corner, the front-left corner and the front-right corner measuring situations, respectively. The measuring results of the terrain drop corresponding to Figure13 are listed in Table 2. The parameters a, b, c, and d of the Table 2, express the four corners: rear-left, rear-right, front-left and front-right, respectively.

From the results, we see the measured test rod altitudes (cm) of the four corners are 84, 108, 101 and 101. The terrain drops of the four corners are −4, −1, −16 and −13. The detected segment length *RA_S_* of the risk area from left-front point to left-rear point, from right-front point to right-rear point, from left-front point to right-front point and from left-rear point to right-rear point, are 107, 104, 352, and 220 cm respectively.

Similarly, the data *RA_f_*_50_, 113, 113, 378, and 224 are the same as the risk area data, and is obtained by moving the camera forward 50 cm, while the data *RA_b_*_50_, 97, 104, 311, 215 is the same as the risk area data, and represents the test results with the camera moving back 50 cm.

**Figure 14.**
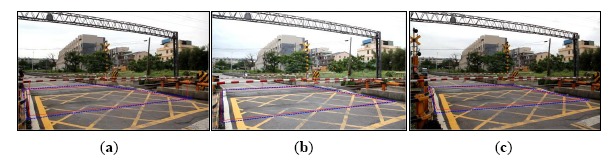
The detection result of the risk area corresponding to case-1 (Doudile Road); the blue-line area is the detection result, and the red-line area expresses the real measured result: (**a**) the measurement result with the camera mounted in the standard position; (**b**) the measurement result with the camera moved forward 50 cm; (**c**) the measurement result with the moved backward 50 cm.

Figure 14 is the detection result of the risk area corresponding to case-1 (Doudile Road); the blue-line area is the detection result, and the red-line area expresses the real measured result. Figure 14a shows the measurement result with the camera mounted in the standard position. Figure 14b shows the measurement result with camera mounted 50 cm forward and Figure 14c shows the measurement result when the camera is mounted 50 cm backwards. If we carefully examine the simulation result and real measured result, we find the difference is little, and it means our scheme has high precision and only a little error.

**Figure 15.**
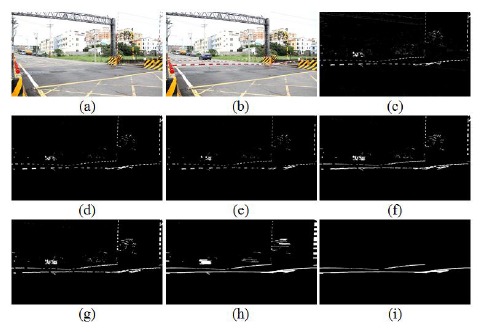
The gates extraction case-2, the railway crossing of NanSikon Road: (**a**) the background image; (**b**) input test image; (**c**) the image after extracting the white part; (**d**) the mage after filtering the noise; (**e**) white part rotation right of (d); (**f**) the image after merging (d) and (e); (**g**) the image after dilation operation; (**h**) the image after the closing operation; (**i**) the image after lengthy fitting operations.

**Figure 16.**
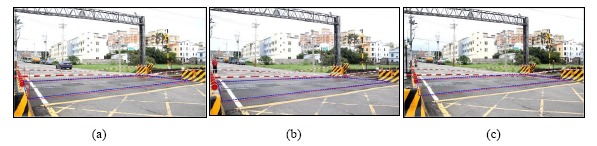
The detection result of the risk area corresponding to case-2, NanSikon Road; the blue-line area is the detection result, and the red-line area is the real measured result: (**a**) the measurement result with the camera mounted in the standard position; (**b**) the measurement result with the camera mounted forward 50 cm; (**c**) the measurement result with the camera mounted 50 cm backward.

**Figure 17.**
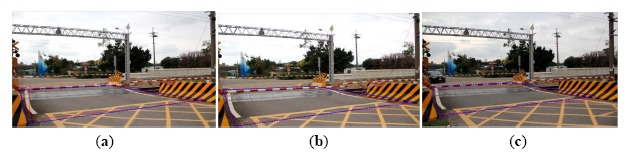
The detection result of the risk area corresponding to case-3, NanPin Road; the blue-line area is the detection result, and the red-line area is the real measured result: (**a**) the measurement result with the camera mounted in the standard position; (**b**) the measurement result with the camera moved forward 50 cm; (**c**) the measurement result with the camera moved backwards 50 cm.

**Figure 18.**
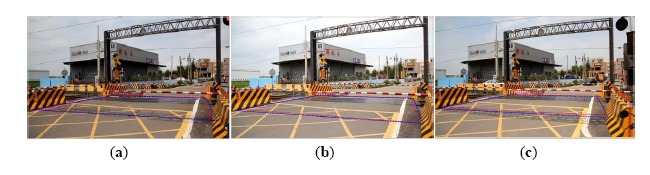
The detection result of the risk area corresponding to case-4, the Danjan Road; the blue-line area is the detection result, and the red-line area is the real measured result: (**a**) the measurement result with the camera mounted in the standard position; (**b**) the measurement result with the camera moved forward 50 cm; (**c**) the measurement result with the camera moved backward 50 cm.

Similary, Figures 15, 16, 17 and 18 are the other test cases; they are taken from NanSikon Road, NanPin Road, and Danjan Road, respectively. They also show high precision and only small errors.

Table 3 lists the measurement results of case-2, where we see the measured test rod altitudes (cm) of the four corners are 82, 93, 87 and 77. The terrain drops of the four corners are −11, −11, −6 and −13. The detected risk area *RA_S_* are 124, 97, 231, 211 cm. Similarly, the data *RA_f_*_50_ are 129, 111, 251, and 221, The data *RA_b_*_50_ are 111, 88, 210, and 204, the same as the risk area data, and these are the results of the camera moving back 50 cm.

On the other hand, Tables 4 and 5 show the test results of case-3 and case-4, respectively. They are similar to case-1 and case-2, and they all gave a high precision and low error. Table 6 lists the error data. The notations *err_S_*, *err_f_*_50_ and *err_b_*_50_ express the test errors of the risk area with the camera in the standard position, camera moving forward 50 cm and camera moving back 50 cm, respectively.

The parameters ‘a’ , ‘b’, ‘c’, ‘d’ are expressed as the measured length difference of the risk area between the real measured and proposed calculated distance from left-front point to left-rear point, from right-front point to right-rear point, from left-front point to right-front point and from left-rear point to right-rear point, respectively. From the above results, it is demonstrated that our scheme is an effective and simple method for railway crossing risk area detection.

**Figure 19.**
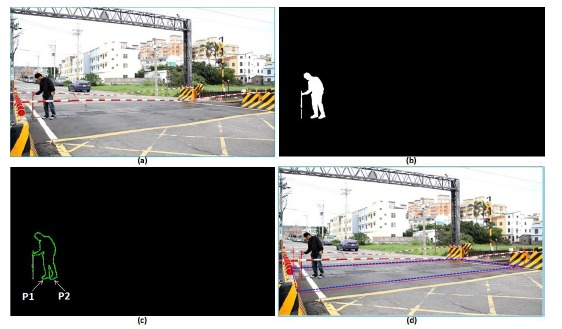
The test image including people taken from NanSikon Road; the blue-line area is the detection result, and the red-line area is the real measured result: (**a**) the original image which is the people are in the railway risk area; (**b**) the people were extracted as an object; (**c**) the edge image of the object; (**d**) the railway risk area detection result.

**Figure 20.**
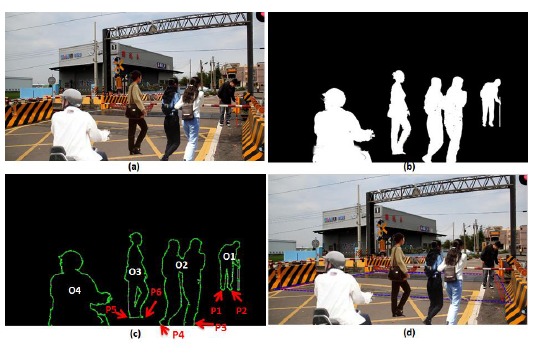
The test image including people taken from Danjan Road; the blue-line area is the detection result, and the red-line area is the real measured result: (**a**) the original image which is the people are in the railway risk area; (**b**) the people extracted as an object; (**c**) the edge image of the object; (**d**) the railway risk area detection result.

Some test images including people were used to simulate the railway risk area detection. Figure 19 taken from NanSikon Road and Figure 20 taken from Danjan Road are the test images which include people in the railway risk area. Figure 19a shows the original image which is the people are in the railway risk area. Figure 19b shows the people extracted as an object in the picture. Figure 19c shows the edge image of the object. It is used to calculate the local Y-coordinate maximum for deciding the ground points of the object. Finally, Figure 19d shows the result of the railway risk area detection. Figure 20 is another test case; there are several people and a motorcycle rider in the test image. From the test results, we see have four objects O1, O2, O3 and O4 that are detected in Figure 20c. We check the local Y-coordinate maximum points p1, p2, p3 …p6, and we find that only p1 and p2 are in the risk area. This means only one person is in the risk area. This makes evident that our scheme can provide a correct detection even in a multi-people situation as shown in Figure 20d.

### 4.3. Function Comparison

In this section, we offer a tabular function comparison between Ku's [4] method and the proposed method. Table 7 shows the comparison results. As its main technique, Ku uses the wireless video communication technique to provide the train driver with a message about the railway crossing situation. On the contrary, our proposed method utilizes the image processing technique; it can automatically detect any intrusion in the risk area. For intrusion detection and preventing accidents, Ku's method depends on people, and it is manual work that easily causes mistakes. Besides, in the risk area error, Ku's method is dependent on people, making it different from the proposed method in that it is limited at 30 cm. As for the response, Ku's method takes less than 15 s and the proposed method is finished in 2 s. We conclude that our method and system can solve the problem automatically, and has high reliability when comparing with Ku's method.

## 5. Conclusions

In this research, we focus on detecting the risk area of railway crossings because most railway accidents happen on railway crossings. In our scheme, we develop a method called white part associated with topology technique (WPAT) to detect the railway crossing gates. Meanwhile, we use a terrain drop compensation (TDC) technique to solve the problem of the concavity of railway crossings. In addition the linear regression technique and image processing technique are used to achieve the goal.

From the simulation results, we obtain correct results when the terrains drop from +20 cm to −27 cm. At the same time, the detection results are also correct when the camera is moved forward 50 cm or moved backward 50 cm. The errors on the Chung-shin Road are 1, 9, 26 and 3 cm for segments a, b, c and d respectively. Similarly, the error data are 5, 11, 28, 11 for Doudile Road, 1, 8, 13, 10 for NanSikon Road, 7, 1, 30, 19 for NanPin Road, and 4, 3, 2, 2 for Danjan Road. It shows the error is limited to 30 cm. It is evident that our scheme is an effective and simple method for the railway crossing risk area detection.

## Figures and Tables

**Table 1.** The results of measuring the terrain drop corresponding to Figure 9.

**Item**	**a**	**b**	**c**	**d**
*l_r_*	84	88	92	85
*l_td_*	21	25	−1	−20
*RA_S_*	80	64	278	117
*err_S_*	1	9	26	3

**Table 2.** The terrain drop measurement results corresponding to Figure 13.

**Item**	**a**	**b**	**c**	**d**
*l_r_*	84	108	101	101
*l_td_*	−4	−1	−16	−13
*RA_S_*	107	104	352	220
*RA_f_*_50_	113	113	378	224
*RA_b_*_50_	97	104	311	215

**Table 3.** The terrain drop measurement results corresponding to case-2.

**Item**	**a**	**b**	**c**	**d**
*l_r_*	82	93	87	77
*l_td_*	−11	−11	−6	−13
*RA_S_*	124	97	231	211
*RA_f_*_50_	129	111	251	221
*RA_b_*_50_	111	88	210	204

**Table 4.** The terrain drop measurement results corresponding to case-3.

**Item**	**a**	**b**	**c**	**d**
*l_r_*	93	101	95	105
*l_td_*	17	13	5	3
*RA_S_*	116	106	325	209
*RA_f_*_50_	115	107	341	220
*RA_b_*_50_	97	85	281	184

**Table 5.** The terrain drop measurement results corresponding to case-4.

**Item**	**a**	**b**	**c**	**d**
*l_r_*	88	93	86	98
*l_td_*	−3	11	−11	6
*RA_S_*	117	100	248	189
*RA_f_*_50_	120	100	265	195
*RA_b_*_50_	115	102	237	190

**Table 6.** The errors of the detection results corresponding to the cases 1–4.

**Items**	**a(cm)**	**b(cm)**	**c(cm)**	**d(cm)**
*err_S_* of case-1	5	11	28	11
*err_f_*_50_ of case-1	1	7	18	1
*err_b_*_50_ of case-1	1	11	28	13
*err_S_* of case-2	1	8	13	10
*err_f_*_50_ of case-2	1	9	15	4
*err_b_*_50_ of case-2	7	11	10	10
*err_S_* of case-3	7	1	30	19
*err_f_*_50_ of case-3	1	2	15	18
*err_b_*_50_ of case-3	11	17	12	29
*err_S_* of case-4	4	3	2	2
*err_f_*_50_ of case-4	5	1	7	10
*err_b_*_50_ of case-4	6	5	18	7

**Table 7.** Function comparison table between the Ku [4] and proposed methods.

**Item**	**Ku [4] Method**	**Proposed Method**
Main technique	Wireless video communication	Image processing
Identification decision	By people	Automatic identification
Intrusion detection	By people (manual work)	By System (Automatic)
Prevent accidents	By people (manual work)	By System (Automatic)
Risk Area Detection Error		≤ 30 cm
Response time	≤ 15 s	≤ 2 s
